# Enhancement of Cortical Network Activity *in vitro* and Promotion of GABAergic Neurogenesis by Stimulation with an Electromagnetic Field with a 150 MHz Carrier Wave Pulsed with an Alternating 10 and 16 Hz Modulation

**DOI:** 10.3389/fneur.2015.00158

**Published:** 2015-07-14

**Authors:** Alexandra Gramowski-Voß, Hans-Joachim Schwertle, Anna-Maria Pielka, Luise Schultz, Anne Steder, Konstantin Jügelt, Jürgen Axmann, Wolfgang Pries

**Affiliations:** ^1^Division of Electrophysiology, NeuroProof GmbH, Rostock, Germany; ^2^Division of Molecular Biology, NeuroProof GmbH, Rostock, Germany; ^3^Engineering Office for Bioresonance and Environmental Technology, Werder/Havel, Germany; ^4^Megawave GmbH, Bad Wörishofen, Germany

**Keywords:** extremely low-electromagnetic field, cortical networks, neurogenesis, multiwell microelectrode recording, phenotypic screening, functional biomarker

## Abstract

In recent years, various stimuli were identified capable of enhancing neurogenesis, a process which is dysfunctional in the senescent brain and in neurodegenerative and certain neuropsychiatric diseases. Applications of electromagnetic fields to brain tissue have been shown to affect cellular properties and their importance for therapies in medicine is recognized. In this study, differentiating murine cortical networks on multiwell microelectrode arrays were repeatedly exposed to an extremely low-electromagnetic field (ELEMF) with alternating 10 and 16 Hz frequencies piggy backed onto a 150 MHz carrier frequency. The ELEMF exposure stimulated the electrical network activity and intensified the structure of bursts. Further, the exposure to electromagnetic fields within the first 28 days *in vitro* of the differentiation of the network activity induced also reorganization within the burst structure. This effect was already most pronounced at 14 days *in vitro* after 10 days of exposure. Overall, the development of cortical activity under these conditions was accelerated. These functional electrophysiological changes were accompanied by morphological ones. The percentage of neurons in the neuron glia co-culture was increased without affecting the total number of cells, indicating an enhancement of neurogenesis. The ELEMF exposure selectively promoted the proliferation of a particular population of neurons, evidenced by the increased proportion of GABAergic neurons. The results support the initial hypothesis that this kind of ELEMF stimulation could be a treatment option for specific indications with promising potential for CNS applications, especially for degenerative diseases, such as Alzheimer’s disease and other dementias.

## Introduction

Already in 1896, Virchow expressed the modern idea in the context of regulation and disease: “The disease begins in the moment when the regulatory apparatus of the body is insufficient to eliminate the interference. Not life under abnormal conditions, not the disorder as such produces disease, but the disease begins with the insufficiency of the regulatory apparatuses” (1). In this respect, an increasing body of literature indicates that electrical and magnetic fields interact significantly with biological systems (2–6). Magnetic and electromagnetic fields (EMFs) are recognized by present-day medicine as real physical entities that could contribute to the healing of various health problems, e.g., pain treatment (7, 8). Electromagnetic therapies are well known and successful in medicine. Proposed and modeled mechanisms are, e.g., ion cyclotron resonance, ion parametric resonance (9, 10), free radicals (11), and the activation of heat shock proteins. One of the first electrochemical models proposed that the cell membrane exerts linear physicochemical changes (12, 13) to assess the EMF parameters for which biological effects might be expected. It was assumed that non-thermal EMF may directly affect ion binding and/or transport, and possibly alter the cascade of biological processes related to tissue growth and repair (4, 6, 14). However, the transduction mechanism for non-thermal EMF biological effects has not been fully elucidated. It is thought that the effectiveness is related to the fine manipulation of cell regulation and communication taking place in this power range.

In recent years, several studies identified various stimuli capable of enhancing neurogenesis in adults, a process, which is dysfunctional in the senescent brain as well as in neurodegenerative and neuropsychiatric diseases. EMFs were already applied in several clinical studies. Saitoh and co-workers demonstrated in a randomized, multicenter, double-blind, crossover, sham-controlled clinical study that daily repeated 5-Hz transcranial magnetic stimulation of primary cortex provided short-term pain relief in neuropathic pain patients (15, 16). They also confirmed that high-frequency transcranial magnetic stimulation over the M1 food area significantly improved motor symptoms in patients with Parkinson’s disease (17). Pries and Baumgart (18) showed in a MRT study that treating patients with knee osteoarthritis twice a day for 60 min for eight consecutive days with exposure to 150 MHz fields pulsed with 10 Hz decreased knee contusion and recovered knee cartilage.

Following up on these reports, we addressed in this study the possible involvement of basic modifications caused by alternating magnetic stimulation by determining functional phenotypic as well as morphological cellular changes. We investigated the effects of repeated exposures to 150 MHz stimulation with pulsed 10 and 16 Hz modulation on the developmental growth of primary cortical networks cultured on microelectrode arrays (MEAs) and their neuronal network activity by an electrophysiological multichannel recording assay. Unlike so-called target-based assays for tests of biological effects on individual receptors or target proteins, functional assays using primary cultures of neural networks on MEAs provide a reflection of the central nervous system with its complex multicellular interconnections. They allow phenotypic screening, which are demonstrably more successful in drug development than target-based approaches (19–22). The MEA neurochip technology is a sophisticated phenotypic high-content screening method to characterize functional changes in network activity in electrically active cell cultures and it is very sensitive to neurogenesis, as well as neuroregenerative and -degenerative aspects (22–28, 29).

## Materials and Methods

### Primary cortical cell cultures

**Figure 1:**
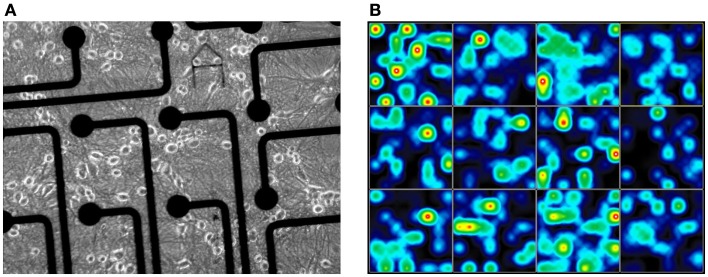
**Multichannel electrophysiological Axion electrode array of the 12-well plate for extracellular recording of cortical network activity**. **(A)** Electrode area with cortical culture at 12 days *in vitro* (scale: 30 μm electrode diameter); **(B)** Spike activity heat map of frontal cortex activity (28 div) cultures in an Axion 12-well MEA with 64 electrodes per well.

Experiments were carried out in accordance with the EU Directive 2010/63/EU on the protection of animals used for scientific purposes (certification file number 7221.3-2). As previously described (26, 30), embryonic frontal tissues were harvested from E 15 NMRI mice (Charles River, Sulzfeld, Germany). Frontal cortex was dissociated enzymatically in DMEM10/10 (10% horse and 10% fetal calf serum) including papain and DNase I, and mechanically with transfer pipettes. The cells were resuspended in DMEM10/10 at a density of 1.0 × 10^6^ cells/ml, and 300 μl were seeded onto each well of 12-well MEA neurochips (Axion Biosystems Inc., Atlanta, GA, USA). Each well contains an array of 64 embedded platinum electrodes resulting in a total of 768 channels. As non-electronic reference 24 well plates with 13 mm cover slips were plated with 100 μl at a density of 1.5 × 10^6^ cells/ml. For better adhesion, the neurochips were coated with poly-d-lysine and laminin before the preparation to ensure cell attachment within a confined adhesive region (5 mm diameter centered around the electrode area). Cultures were kept at 37°C in a 10% CO_2_ atmosphere for 4 weeks and fed twice a week with DMEM containing 10% horse serum (Figure 1A). The developing co-cultures were treated at day 5 *in vitro* with 5-fluoro-2′-deoxyuridine to prevent glial proliferation and overgrowth.

### Alternating magnetic field stimulation

**Figure 2:**
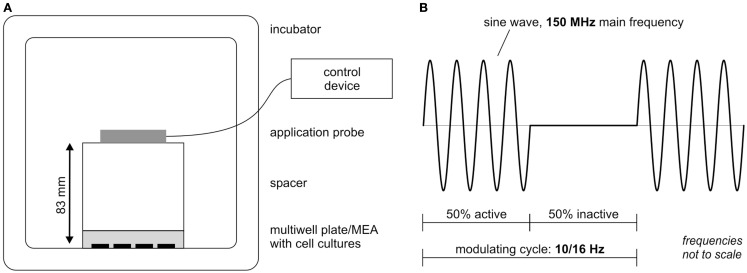
**Extremely low-electromagnetic field exposure set up scheme**. **(A)** Experimental stimulation set up in the incubator. **(B)** Waveform scheme of the extremely low-electromagnetic field studied here: a 150-MHz carrier frequency signal is pulse modulated with 50% time modulation burst (100 or 62.5 ms) repeated for modulating frequencies 10 and 16 Hz.

The extremely low electromagnetic field (ELEMF) signal used in this study was generated by the stimulation device MegaWave MW150 (MegaWave GmbH, Bad Wörishofen, Germany), which is an accredited medicine product according to the medicine product guideline 93/42/EWG. It has a high-frequency oscillator stage that generates a fixed carrier frequency of 150 MHz. A low-frequency oscillator stage generates an adjustable lower frequency as the modulation frequency. Settings used in this study are depicted in Figure 2. The stimulation parameters as well as the distance between cortical cultures and stimulation probe were chosen from pilot study data (data not shown). The durations of the cycles were set to 100 or 62.5 ms (equivalent to 10 or 16 Hz) with modulation setting to 50% cycle time. The 50% stimulation rhythm (50 or 31.25 ms) was followed by 50% silence phase (50 or 31.25 ms) (Figure 2B). The power level was set to 3 resulting in a power output of 0.240 mW ± 15%. The magnetic induction pulses at the applied settings equaled a maximum magnetic flux density of 0.125 pT (125 E-15 Vs/m^2^). The distance between the applicator probe (cross section diameter 70 mm) and the cortical cultures was set to 83 mm (Figure 2A). Plastic ware empty multiwell plates were used as spacer. The cortical network cultures on Axion 12-well MEAs and 24-well plates were successively exposed from 4 to 28 days *in vitro* twice a week in a designated incubator under constant conditions (10% CO_2_, 37°C) first to 30 min of a modulating frequency of 10 Hz followed by 30 min at 16 Hz. The control group (separate Axion 12-well MEAs and 24-well plates) was handled with the same procedure, except for the EMF exposure.

### Multichannel recording

In principle, electrophysiological studies were performed as described by Gramowski et al. (26). Recordings were executed with the Maestro recording system by Axion Biosystems Inc. (Atlanta, GA, USA) providing 1200× amplification, sampling at 12.5 kHz, filtering, and spike detection, delivering whole channel neuronal spike data. For extracellular recordings, MEAs were maintained at 37°C and a pH of 7.4 through a continuous filtered and humidified airflow with 10% CO_2_. Recordings were made in DMEM with 10% horse serum. The network response was recorded for at least 1 h (Figure 1B). The neuronal networks were recorded after 14 and 28 days *in vitro*, respectively.

### Multiparametric data analysis

Each unit represents the activity originating from one neuron recorded at one electrode. Units are separated at the beginning of the recording. We analyzed the stable activity phase of the last 30 min. Action potentials (spikes) were recorded as spike trains, which are clustered in so-called bursts. Bursts were quantified via direct spike train analysis using the standard interspike interval (ISI) method in NPWaveX (NeuroProof GmbH, Rostock, Germany). Bursts are defined by the following parameters: maximum ISI to start a burst: 40 ms, minimum ISI to end a burst: 200 ms, minimum interval between bursts: 100 ms, minimum duration of burst: 10 ms, and min number of spikes in a burst: 2.

With a multiparametric high-content analysis of the network activity patterns, we extract 204 activity-describing spike train parameters and thereby obtain a precise description of activity changes in the four categories as follows: general activity, burst structure, synchronicity, and oscillatory behavior. Changes in general activity parameters describe the effects on action potential firing rate (spike rate), burst rate, and burst period as the time between the bursts. Burst structure parameters define not only the internal structure of spikes within a high-frequency spiking phase (burst), e.g., spike frequency in bursts, spike rate in bursts, and burst spike density, but also the overall structure of the burst, such as duration, area, and plateau. Oscillatory parameters quantify the regularity of occurrence or structure of bursts, which is calculated by coefficients of variation of primary activity parameters describing the variability of parameters (general activity, burst structure) within experimental episodes (31). Higher values indicate less regular burst structure or less regular general activity (e.g., spiking, bursting). As a measure of synchronicity in the spike trains, CVnet parameters reflect synchronization among neurons within the network (31). CVnet is the coefficient of variation over the network. Large CVnet values imply a wide range of variation in the activity across the network, meaning less synchronization. Twelve out of 204 parameters were visualized as the most describing ones divided into the four categories as follows: general activity, burst structure, oscillation, and synchronization. The definitions for these 12 parameters are as follows: spike rate: number of spikes per second, averaged over all spike trains recorded per 60 s bin; burst rate: number of bursts per second, a measure for burstiness of units, averaged over all units recorded per 60 s bin; spike contrast: describes the occurrence or absence of spikes in neighboring time segments of the spike train, reflecting the variability in burstiness of units within experimental episodes; burst duration: mean lengths of bursts (milliseconds) based on ISI method; burst amplitude: bursts are mathematically superimposed with an integral function. The integral is defined by spike peak density in bursts and number of spikes. Burst amplitude is the peak amplitude of the integrated burst reflecting the fraction of the bursts with highest spike density; burst spike density: mean frequency of spikes within bursts (Hertz), defined by the average of all interspike intervals in a burst. Burst spike density increases if the number of spikes in burst increases or burst duration decreases; burst rate SD: SD of burst rate across 60 s bins, indicating the variability of burstiness of units within temporal episodes; Syn All: average distance of bursts within a population burst from population burst center, a measure for the strength of synchronicity of a network; Syn share: average fraction of units involved in population bursts, with higher values reflecting a higher degree of synchronicity between the units; burst rate CVnet: coefficient of variation of burst rate, reflecting spatial variation of burst rate over the network during experimental episodes; event rate: number of burst events per second, with an event defined as synchronous burst activity of at least 50% of all units in a network within a time frame of 300 ms; hamming factor: or hamming distance between two spike trains is the number of positions at which corresponding burst events are different. Here, the hamming factor is calculated for all corresponding spike trains with a binning of 10 s.

### Cross validation and similarity analysis

Training data sets with all 204 spike train parameters were calculated for control and treatment groups. The respective data records of the two groups (exposure treatment and control) were classified using methods of pattern recognition (software package PatternExpert, NeuroProof GmbH, Rostock, Germany). As previously describes (26, 32), an artificial neuronal network was trained with the data using a multi-layer feed forward perceptron neural network and back propagation algorithm without hidden units and a resilient-propagation learning algorithm. There were as many input knots as there were parameters (204) and 4 output nodes, 1 for each classified group. Non-usage of hidden layers was justified by the relatively high variation of the data. The performed cross-validation indicates the functional similarity/dissimilarity of the treatment group and the control. χ^2^ analysis was performed on the cross-validation data to test for significant effects.

### Immunocytochemistry

The recorded cortical cultures on Axion 12-well MEAs, as well as concurrently cultured cortical networks on 24-well plates with 11 mm glass cover slips from the different treatment groups were further analyzed by immunocytochemistry, fluorescence microscopy, and semi-automatic quantitative image analysis. The cells were initially washed with PBS and fixed with 4% paraformaldehyde for 30 min, followed by the addition of a PBS-based blocking solution containing 1% BSA, 2% goat serum, and 0.05% TWEEN20. Cells were immunostained with primary antibodies for neurites with anti-tubulin beta-III (1:750; Sigma-Aldrich, Germany), neuronal soma with anti-Hu C/D (1:500; neuronal-specific RNA-stabilizing protein present in neuronal cell bodies) (Invitrogen, Germany), synapses with anti-synapsin-1 (1:200; Cell Signaling, Germany), GABAergic neurons with anti-GABA (1:500; Sigma-Aldrich), and nuclei with the DNA dye Hoechst/Bisbenzimide (1 μg/ml; Sigma-Aldrich, Germany). Cortical networks were embedded with Prolong antifade gold (LifeTechnologies, Germany) and analyzed with an upright fluorescence microscope (Nikon Eclipse E800, Nikon AG, Japan). Images were analyzed using a semi-automatic image quantification tool (ImageJ2x, Rawak Software, NIH, USA). The following parameters were quantified per image: cell number, neuron number, neurite number, synapse number, % neurons, neurites per neuron, synapses per neurite, and number of GABAergic neurons.

### Statistical analysis

For the data and statistical analysis, all recorded networks in each treatment group were used with no exception.

Results from primary analysis are expressed as series means ± SEM. The absolute parameters’ distributions were tested for normality. The level of significance after exposure treatment was assessed by using Student’s *t*-test and *p* < 0.05 was considered statistically significant.

## Results

**Figure 3:**
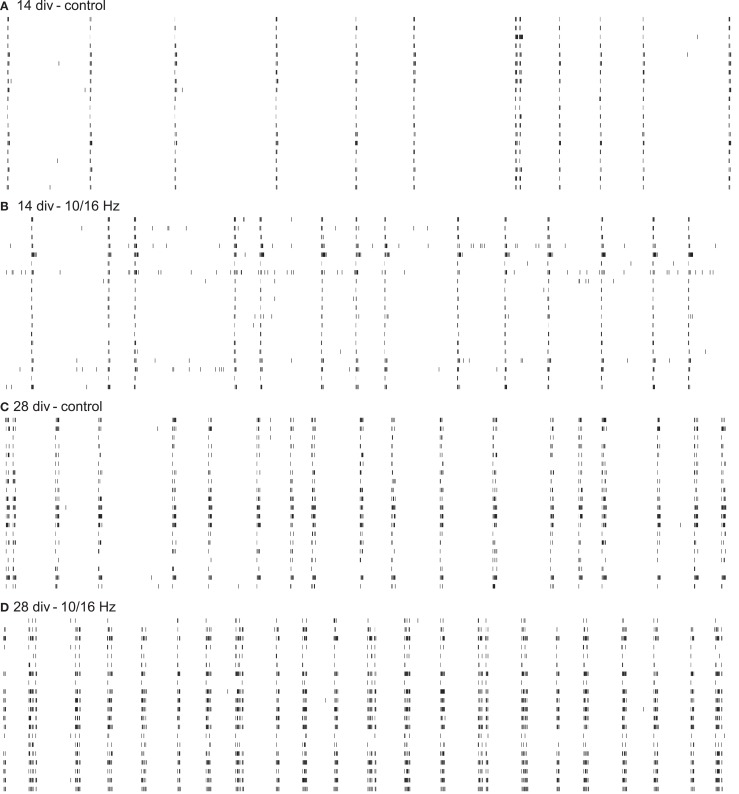
**Cortical spike trains of 60 s from 20 neurons within one network depict the functional electrophysiological effects of 150 MHz electromagnetic field exposure modulated with pulses of extremely low frequencies of 10 and 16 Hz on the differentiation of the cortical network activity**. Increase in spike train activity during network maturation **(A,C)**; enhancement of spike train activity during development by ELEMF stimulation **(B,D)**.

In this study, we investigated the effects of electromagnetic exposure of an ELEMF with a 150 MHz carrier frequency, which was alternatingly modulated with 10 and 16 Hz, on the differentiation of cortical networks’ activity and morphology after 10 (14 div) and 24 days (28 div) of exposure. Therefore, we recorded the network activity as spike trains (Figure 3) and stained the cortical cultures for various morphological markers. We compared the effects of exposed and control condition.

### Developmental changes of the cortical network activity and morphology under control conditions

**Figure 4:**
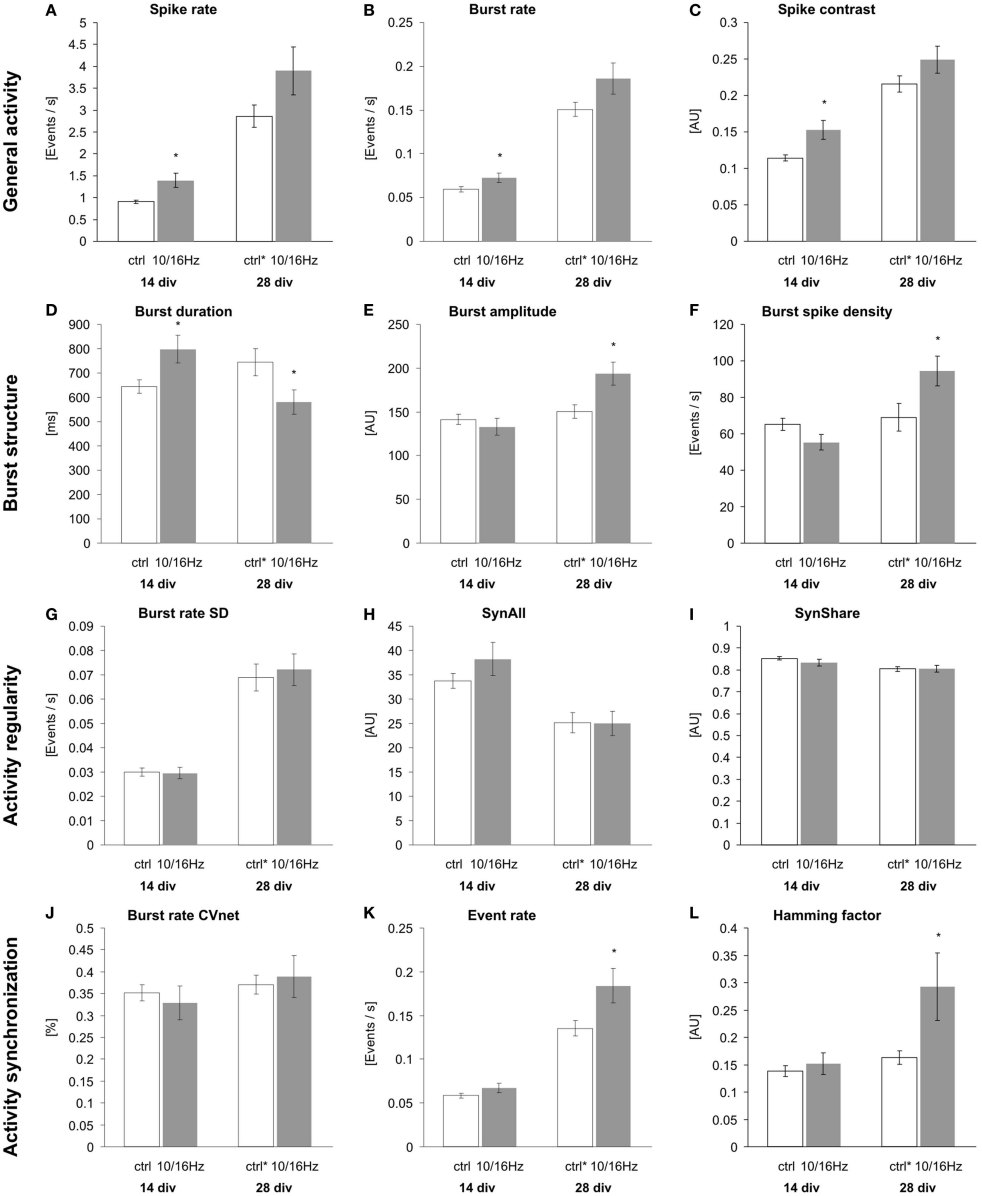
**Effects of 150 MHz electromagnetic field exposure pulse modulated with alternating 10 and 16 Hz on the differentiation of cortical network activity**. Plotted are 12 activity-describing parameters from the multiparametric data analysis. The extracted parameters reflect the changes in the four activity categories general activity **(A–C)**, burst structure **(D–F)**, synchronization **(G–I)**, and oscillation **(J–L)**. Statistical significance is shown for the comparison between the control and the ELEMF group (mean ± SEM, Student’s unpaired*t*-test: *p ≤ 0.05; **p ≤ 0.01; ***p ≤ 0.001).

Under control conditions, the development of the cortical network activity was characterized by a boost in general activity (Figures 3A,C). Here, from 14 to 28 div, the overall spiking and bursting activity increased approximately 3- and 2.5-fold. This is revealed by the increase in spike rate, burst rate, and the “spike contrast” to 317, 253, and 189%, respectively (Figures 4A–C). This activity enhancement was accompanied by a slight enlargement of burst structure with an increase in burst duration, burst amplitude, and burst spike density to 130, 106, and 106%, respectively (Figures 4D–F). The network pattern regularity remained unchanged during activity development (Figures 4A–C), as revealed by the unchanged spike and burst rate coefficients of variations over time (CVtime, data not shown). The increase in the spike rate SD was accompanied by an increase of the spike rate itself and the increase of the number of units recorded. The number of bursting units increased by 100% from 6 ± 1 to 12 ± 2 bursts per minute. A similar development was found for parameters reflecting the spiking and bursting synchronicity. The burst rate variation over the network (CVnet values) and the hamming factor were unaffected from 14 to 28 days (Figures 4J–L). Only the event rate increased over the network maturation to 213% due to the general increase in the spike and burst rate.

**Figure 5:**
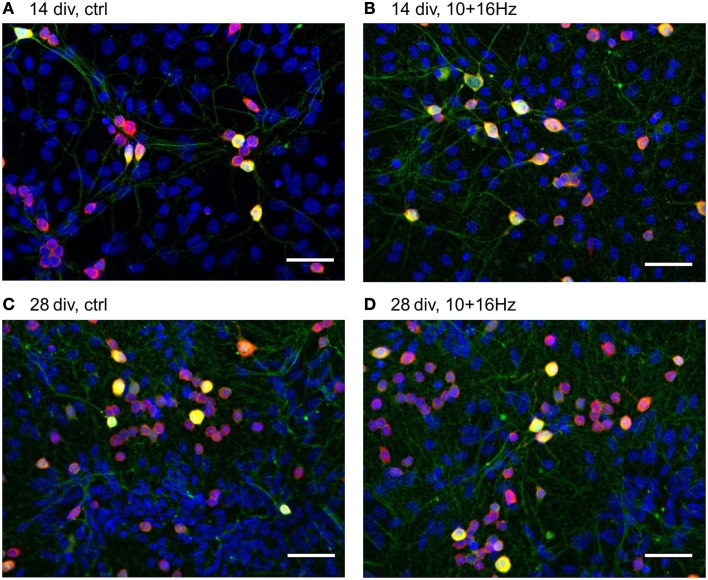
**Effects of 150 MHz electromagnetic field exposure pulse modulated with alternating 10 and 16 Hz on the development of cortical network morphology**. Increase of total cell number, neurons, GABAergic neuronal subpopulation, and neurites during development from 14 to 28 div **(A,C)**; Additional increase in neurites and shift towards the neuronal population by ELEMF stimulation at 14 div **(B)** and shift to the GABAergic neuronal subpopulation by ELEMF stimulation at 14 and 28 div **(B,D)**. Color-merge of GABAergic neurons (green, anti-GABA), neuronal soma (red, anti-HuC/D), nuclei (blue, Hoechst), bar = 50 μm.

**Figure 6:**
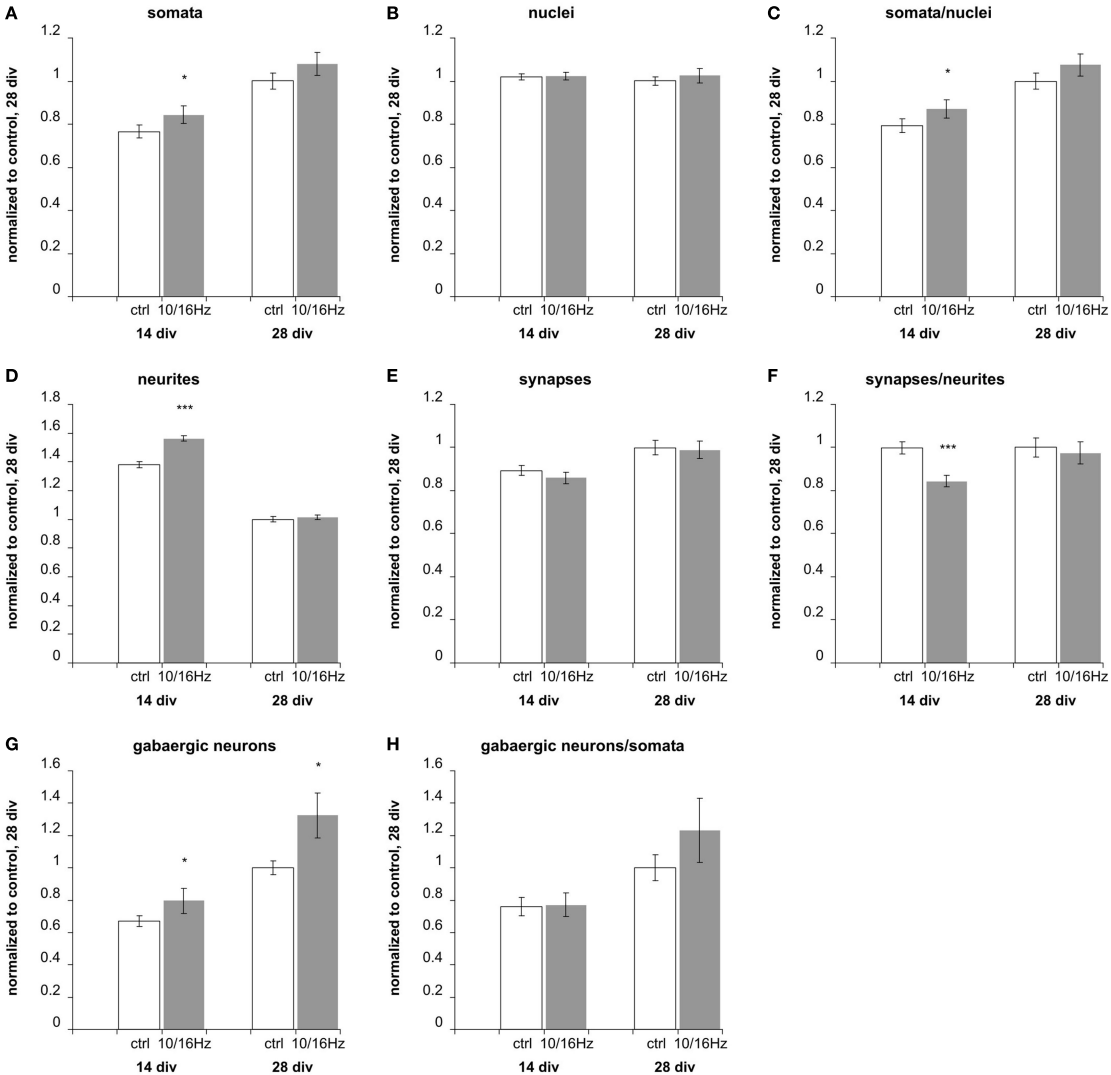
**Effects of 10 and 16 Hz pulse modulated 150 MHz electromagnetic field exposure on the development of cortical network morphology**. Semi-automatic quantification of neurons/image, total cell nuclei/image, neurites/image, and synapses/image and extraction of the neuronal marker: **(A)** total cell number, **(B)** number of neurons, **(C)** percentage of neurons, **(D)** number of neurites, **(E)** number of synapses, **(F)** synapses per neurite, **(G)** number of GABAergic neurons, **(H)** percentage of GABAergic neurons of neurons. Statistical significance is shown for the comparison between the control and the ELEMF group (mean ± SEM, Student’s unpaired *t*-test: *p ≤ 0.05; **p ≤ 0.01; ***p ≤ 0.001).

This cortical network activity enhancement was accompanied by changes in the morphology of the cortical neuron/glia co-culture (Figure 5). During the differentiation of the cortical networks between 14 and 28 div, the percentage of neurons increased from 19 ± 2 to 24 ± 2%, whereas the total number of cells remained unaltered (Figure 6). The neurite density decreased by 29%. In addition, the percentage of GABAergic neurons increased from 19 ± 2 to 25 ± 3% (Figure 6).

### Electromagnetic 150 MHz exposure with 10 and 16 Hz modulation increased the cortical network activity during the process of network activity maturation

At 14 div, after four treatments over the course of 10 days, the electromagnetic exposure caused an acceleration in the development of the network activity (Figures 3A,B). This is demonstrated by the increase in the general activity parameters spike rate, burst rate, and spike contrast to 155, 122, and 133%, respectively (Figures 4A–C). These general activity changes were accompanied by burst structure changes characterized by an elongation in burst duration to 130%, compared to control levels at 14 div (Figures 4D–F). The regularity and synchronicity of the network activity were not affected (Figures 4G–L).

The prolonged electromagnetic exposure (eight treatments over 24 days) over the cortical activity differentiation period of 28 days *in vitro*-induced activity changes of the same trend. The general activity was increased with respect to spike rate, and burst rate, and the spike contrast to 136, 123, and 116%, respectively. However, it was accompanied by reorganization within the burst structure (Figures 3C,D). While the burst duration was decreased to 81%, burst amplitude and burst spike density were increased to 129 and 137%. Also, the network patterns’ synchronicity was increased, quantified by the increase in the event rate and the Hamming factor to 115 and 179%, respectively. Again, the network pattern regularity was not affected (Figure 4).

To further underpin the obtained results, all multiparametric spike train data sets of control and exposure groups at 14 and 28 div were further analyzed using pattern recognition. The cross-validation results of the classification revealed a good self-recognition of each data group and separation from the respective treatment or control groups (Table 1). About 76 and 71% of all data sets for 14 and 28 div cortical network activity recordings, respectively, recognized themselves correctly as control. In the ELEMF exposure groups, 56 and 59% of all data sets recognized themselves correctly and significantly as exposure group. Here, the diversity in percentage of correct recognition is due to the different size of data sets (=experiments per group) in the four groups. The χ^2^ test, which considers the expectation value for each pattern recognition test, indicates significant effects.

**Table 1:** **Cross validation of exposure treatment group vs. control group: the activity profiles of the treatment groups at 14 and 28 div separate significantly from their respective control groups**.

Day of recording	Data group	Control (%)	10 **+** 16 Hz (%)	**χ**^2^ test (*p* value)
14 div	Control	76	24	
	10 + 16 Hz	44	56	0.0028
28 div	Control	71	29	
	10 + 16 Hz	41	59	0.0068

*Values correspond to % of datasets of one group (rows) classified as either itself or the other group. The higher this value, the more distinct the functional profile is from each other. The statistical verification is performed by χ^2^ test showing the separation of the datasets is statistically significant*.

### Effects of electromagnetic exposure on the development of the cortical network morphology

The repeated electromagnetic exposure with alternating 10 and 16 Hz frequency did not only influence the cortical network activity but also affected the development of the cortical network morphology (Figure 5). For a closer investigation of the mode of action, how the electromagnetic exposure changed the functional activity by either supporting neurogenesis, neuritogenesis, or synaptogenesis, the chronically treated cortical cultures on MEAs were fixed after recording to assess the cellular effects by immunocytochemistry. We quantitatively assessed synaptogenesis (synaptic puncta per image, per neuron), neuritogenesis (number of neurites/100 μm per image, per neuron), and neurogenesis (% neurons compared to non-neuronal cells), as well as the portion of GABAergic neurons by immunostaining for specific markers.

At 14 days *in vitro*, 10 days of repeated exposure induced an accelerated differentiation of cells into neurons, shown by an increase of neuronal somata by 11% with an unaltered total number of cells and accompanied increase of their ratio by 10% (Figures 6A–C). This is also underlined by an enhanced outgrowth of neurites to 113% (Figure 6D). The number of total synapses remained unaltered (Figure 6E), resulting in a decrease of the synaptic density (number of synapses per neurite) to 85% (Figure 6F). The staining of GABAergic cells revealed an increase in this specific neuronal population (Figure 4G) to 119%, while the unaffected ratio of GABAergic to total neuronal cell number (Figure 4H) indicates that the exposure induced an enhanced differentiation of a GABAergic neuronal subpopulation during early *in vitro* cortical cell culture differentiation.

These early effects of the alternating 10 and 16 Hz electromagnetic field exposure were largely compensated after 24 days of repeated exposure at div 28 (Figure 6). At this culture age, the differentiation is assumed to be completed; only the GABAergic neuronal subpopulation persisted at an increased level of 132% (Figure 6G).

To evaluate potential secondary effects of the ELEMF due to the platinum electrodes and conducting path in the MEA neurochip, we additionally analyzed the effects of the ELEMF stimulation of cortical networks on cover slips in 24-well plates. We chose 24-well over 12-well plates because their geometries provide culture conditions similar to the Axion 12-well MEAs considering cell density, total number of cells seeded, seeding area, and medium volume. The cortical networks in 24-well plates revealed an increase of total number of neurons by 19% and an increase in the neuronal percentage by 22%. Here, the GABAergic population of neurons increased by 24% after 24 days of the ELEMF stimulation. These data are comparable to those obtained on the Axion 12-well chip. Hence, the effects were slightly higher than on the MEA chips, suggesting particular small counter effects induced by the electronics (electrodes and conducting paths) on the MEA chip.

## Discussion

We demonstrated that the exposure of primary cortical *in vitro* networks with a 150 MHz carrier frequency, pulse modulated with alternating 10 or 16 Hz increased activity and intensified the burst structure within network activity. Overall, this effect was most pronounced already after 10 days of exposure at div 14. The development of cortical activity under these conditions was accelerated. These functional changes were accompanied by morphological ones. The effects on the morphological changes induced by the ELEMF stimulation on cortical networks in corresponding 24-well plates were higher compared to the effects induced in the networks on 12-well Axion chips. Yet, the percentage of neurons was increased with an unaffected total number of cells. This indicates an enhancement of neurogenesis or neuronal differentiation. The increase in neurites accompanied with a decrease of the synaptic density per neurites in the exposure group at 14 div also indicates a prolonged synaptic plasticity by the ELEMF exposure. The electromagnetic stimulation selectively promoted the differentiation of neuronal populations, evident by the increased proportion of GABAergic neurons, which sustained a higher ratio after 24 days of stimulation at div 28. In contrast to studies where repeated pentylenetetrazole (PTZ) stimulation resulted in an increase in migrating neurons in hippocampus and induced seizure network activity (33, 34), the recordings of our cortical network activities showed no typical seizure activity at neither 14 nor 28 div.

In Alzheimer’s disease (AD), numerous neurofibrillary tangles and Aβ plaques occur, and neurogenesis and neuronal plasticity are also markedly altered (35).

Therefore, much research effort has been devoted to identify stimuli capable of enhancing adult neurogenesis. Researchers provide evidence for one desirable outcome that can be attained via suitable magnetic fields, demonstrating an effective boost to rates of neurogenesis, the creation of new neurons in the brain. Higher rates of neurogenesis imply greater neuroplasticity, the ability of the brain to adapt, repair, and resist minor damage, which overall seems to be a good target to aim for.

Comparable to our *in vitro* approach the group around Adey demonstrated that ELEMF exposure (147 MHz, 6–20 Hz amplitude modulation) reinforced spontaneous brain rhythms in cats (36) as well as increasing the calcium efflux from chick cerebral tissue by more than 15% (37, 38). Due to the very high frequency of 150 MHz and the associated extreme slew rate of the device’s pulse signals such a low value can still induce relatively high voltages (millivolts to 1 V) and currents (100 μA to 1 mA) in tissues or cells, which in turn may also have relevant physiological effects on the membrane potential or ionic currents of voltage-sensitive ion channels. Piacentini et al. (39) reported an increased expression and function of voltage-gated Ca^2+^ channels after exposure to ELEMFs whereat the Ca^2+^ influx through Ca(v)1 channels played a key role in promoting neuronal differentiation of murine neural stem/progenitor cells *in vitro*. In mice, ELEMFs *in vivo* promoted the proliferation and neuronal differentiation of hippocampal neural stem cells that functionally integrate in the dentate gyrus (40, 41) and enhanced newborn neuron survival *in vivo* (3.5 h/day for 6 days) (42). These alterations were associated with enhanced spatial learning and memory. Extremely low-frequency electrical or magnetic fields are thought to evoke changes in neuronal membrane potential at most in the microvolt range, which at first sight appear orders of magnitude too small to significantly influence neuronal signaling. However, a number of mechanisms in the central nervous system can amplify these signals, potentially allowing such small changes in membrane potential to induce significant physiological effects [for review, see Ref. (43)]. It has been reported that exposure to weak extremely low-frequency (ELF, 1–100 Hz) magnetic fields can influence brain electrical activity (EEG) in humans and animals (44, 45). Many behavioral effects have been reported for exposure to magnetic fields at different intensities, particularly with pulsed magnetic fields. The specific pulse form appears to be an important factor for the behavioral effects seen; for example, a pulsed magnetic field originally designed for spectroscopic MRI was found to alleviate symptoms in bipolar patients (46), while another MRI pulse had no effect. A whole-body exposure to a pulsed magnetic field was found to alter standing balance and pain perception in other studies (47, 48).

Given the role of stem cells, e.g., in hippocampal functions, such as cognition, and given their potential for brain repair, Fitzsimons et al. (49) reviewed the epigenetic mechanisms relevant for neuron stem cell proliferation and AD etiology. In this context, Robel et al. (50) discussed a new view on astrocyte-like reactive glia, as a suggested widespread endogenous source of cells with stem cell potential, which might potentially be harnessed for local repair strategies. Ma et al. (51) argued for a role of these glia cells in controlling multiple steps of adult neurogenesis, from proliferation and fate specification of neural progenitors to migration and integration of the neuronal progeny into pre-existing neuronal circuits in the adult brain and their potential function in degenerative neurological diseases, such as AD. These data are consistent with data presented here, where the ELEMF exposure-induced neurogenesis without affecting the total number of cells, which suggests that progenitor cells where differentiated into neurons instead to glial cells. Such enhanced differentiation into neurons seems to be directed into the GABAergic neuron population. This could be important, because precisely timed neuronal activity across brain regions is crucial for cognitive processing (52) and, e.g., a division of GABAergic cell types is controlling network activities in hippocampus (53–55). Those GABAergic cells in a cell-type specific manner coordinate brain rhythm interactions. A slight change in the ratio of GABAergic cell types could result in diverse changes of electrical activity patterns in our cortical cultures.

Another hypothesis is that the ELEMF exposure could trigger alpha frequency bands in the brain, and induce similar changes as confirmed for neurofeedback training of the upper-alpha frequency band, improving cognitive enhancement (56–58). Rhythmic activity in the alpha band (8–13 Hz) is a core feature of cortical communication (59) and cognition (60) and was demonstrated in various studies to modulate neural firing (61), perceptual detection rates (62, 63), and cerebral blood flow (64). Alpha-band oscillations exert a critical influence on the excitability level of neocortical networks as well as on the timing of neuronal responses (59, 65). Alpha rhythms were also observed in thalamic neurons *in vitro* (66, 67). Electrophysiological *in vitro* recordings and computational models of thalamic circuits show that during strong phasic inhibition the spiking of relay-mode neurons and pyramidal cells are entrained by the phase of the alpha cycle (68, 69). Upper-alpha frequency is widely shown to be correlated with cognitive performance (70, 71). First clinical trials with the here applied ELEMF frequency exposure design on patients with mentally impaired perception revealed an improvement of their cognitive behavior after daily stimulation with 150 MHz with a pulsed 10 and 16 Hz modulation for 14 days; in this respect, in an ongoing clinical study, the effects of the ELEMF stimulation on diminution of beta-Amyloid deposition are tested [personal communication Prof. W. Pries].

In conclusion, the studies discussed above support the initial hypothesis that this kind of stimulation is not only a treatment option for specific diseases or indications but also a completely new therapeutic low-energy electromagnetic field approach with promising potential for CNS applications. Further detailed studies to elucidate the molecular mechanisms mediating the ELEMF effects might lead to the development of novel therapeutic approaches for degenerative diseases, such as AD.

## Conflict of Interest Statement

This work was financially supported by MegaWave GmbH, Germany.

## Abbreviations

AD, Alzheimer’s disease; CNS, central nervous system; CV, coefficient of variation; Div, days *in vitro*; DMEM, Dulbecco’s modified Eagle’s medium; EEG, electroencephalography; ELEMF, extremely low-electromagnetic field; GABA, gamma-aminobutyric acid; MEA, microelectrode array; MRI, magnetic resonance imaging; NIH, National Institute of Health; PTZ, pentylenetetrazole; SEM, standard error of mean.
